# Induction of IFN-β and the Innate Antiviral Response in Myeloid Cells Occurs through an IPS-1-Dependent Signal That Does Not Require IRF-3 and IRF-7

**DOI:** 10.1371/journal.ppat.1000607

**Published:** 2009-10-02

**Authors:** Stephane Daffis, Mehul S. Suthar, Kristy J. Szretter, Michael Gale,, Michael S. Diamond

**Affiliations:** 1 Department of Medicine, Washington University School of Medicine, St. Louis, Missouri, United States of America; 2 Department of Immunology, University of Washington School of Medicine, Seattle, Washington, United States of America; 3 Department of Molecular Microbiology, Washington University School of Medicine, St. Louis, Missouri, United States of America; 4 Department of Pathology & Immunology, Washington University School of Medicine, St. Louis, Missouri, United States of America; Cleveland Clinic, United States of America

## Abstract

Interferon regulatory factors (IRF)-3 and IRF-7 are master transcriptional factors that regulate type I IFN gene (IFN-α/β) induction and innate immune defenses after virus infection. Prior studies in mice with single deletions of the IRF-3 or IRF-7 genes showed increased vulnerability to West Nile virus (WNV) infection. Whereas mice and cells lacking IRF-7 showed reduced IFN-α levels after WNV infection, those lacking IRF-3 or IRF-7 had relatively normal IFN-b production. Here, we generated IRF-3^−/−^× IRF-7^−/−^ double knockout (DKO) mice, analyzed WNV pathogenesis, IFN responses, and signaling of innate defenses. Compared to wild type mice, the DKO mice exhibited a blunted but not abrogated systemic IFN response and sustained uncontrolled WNV replication leading to rapid mortality. Ex vivo analysis showed complete ablation of the IFN-α response in DKO fibroblasts, macrophages, dendritic cells, and cortical neurons and a substantial decrease of the IFN-β response in DKO fibroblasts and cortical neurons. In contrast, the IFN-β response was minimally diminished in DKO macrophages and dendritic cells. However, pharmacological inhibition of NF-κB and ATF-2/c-Jun, the two other known components of the IFN-β enhanceosome, strongly reduced IFN-β gene transcription in the DKO dendritic cells. Finally, a genetic deficiency of IPS-1, an adaptor involved in RIG-I- and MDA5-mediated antiviral signaling, completely abolished the IFN-β response after WNV infection. Overall, our experiments suggest that, unlike fibroblasts and cortical neurons, IFN-β gene regulation after WNV infection in myeloid cells is IPS-1-dependent but does not require full occupancy of the IFN-β enhanceosome by canonical constituent transcriptional factors.

## Author Summary

West Nile virus (WNV) is a mosquito-transmitted virus that infects birds, horses, and humans and has become an emerging infectious disease threat in the Western Hemisphere. In humans, WNV can invade into the brain and spinal cord and destroy neurons, causing severe neurological disease, particularly in the immunocompromised and elderly. A better understanding of how the immune system controls WNV infection is critical for developing new treatments and vaccines. In this study, using a mouse model of WNV infection, we evaluate the combined role of two key transcription factors, interferon-regulatory factor-3 (IRF-3) and IRF-7, that orchestrate antiviral and interferon (IFN) responses after infection. Mice that lack both IRF-3 and IRF-7 were highly vulnerable to lethal infection and cells lacking IRF-3 and IRF-7 had a markedly attenuated IFN-α response. Surprisingly, macrophages and dendritic cells lacking IRF-3 and IRF-7 showed a relatively normal IFN-β response. Furthermore, a genetic deficiency of IPS-1, a protein that signals downstream of the RIG-I- and MDA5 cytoplasmic viral RNA sensors, completely abolished IFN-β production. Our experiments suggest that in specific cell types infected with WNV, IFN-β can be induced through an IPS-1-dependent transcriptional signal that does not require the master transcriptional regulators IRF-3 and IRF-7.

## Introduction

The rapid production of type I interferon (IFN-α/β) and the IFN-induced antiviral response serve as primary host defense mechanisms against infection by many viruses (reviewed in [1]–[3]). IFN-α/β gene transcription is induced after host pattern recognition receptors (PRR) bind pathogen-associated molecular patterns (PAMP), such as viral nucleic acids (reviewed in [4]–[6]). A current paradigm for type I IFN production after RNA virus infection describes a two-step or positive feedback model that is modulated by the master transcription factors interferon regulatory factors (IRF)-3 and -7 [7]–[9]. In the initial phase, viral sensing by PRR induces nuclear localization of IRF-3, which stimulates gene transcription and production of IFN-β and IFN-α4 by infected cells. In the second phase, these IFNs bind to a common IFN-α/β receptor in a paracrine and autocrine manner and signal through the JAK-STAT pathway resulting in the induced expression of hundreds of interferon stimulated genes (ISG) (e.g., PKR, RNAse L, viperin, ISG15, and ISG20), which limit viral replication through multiple mechanisms [10]–[13]. Whereas IRF-3 is constitutively expressed throughout many tissues and functions downstream of specific PRR (e.g., TLR3, MDA5, and RIG-I) to inhibit RNA viruses, IRF-7 is both an ISG and a transcriptional activator downstream of distinct PRR (e.g., TLR7 and TLR8); IRF-7 participates in an IFN amplification loop by inducing IFN-β and many subtypes of IFN-α [14].

Recognition of West Nile virus (WNV), a neurotropic virus of the *Flaviviridae* family of RNA viruses, by the intrinsic cellular immune response is believed to occur through concerted signals by several PRR (TLR3, TLR7, TLR8, RIG-I, and MDA5) that recognize single or double-stranded RNA and signal through their constituent adaptor molecules (TRIF, MyD88, and IPS-1). Tissue culture experiments with murine embryonic fibroblasts (MEF) suggested that RIG-I is an initial and primary PRR for WNV as genetically deficient cells had an abrogated ISG response at early time points after infection [15]. The late ISG response appears more dependent on MDA5, suggesting a dual requirement of both RIG-I and MDA5 for activation of an effective cellular antiviral response against WNV, at least in MEF [16]. In contrast, a deficiency of TLR3 in MEF, macrophages, and dendritic cells did not alter IFN-α/β production or viral burden, indicating a more limited role of this sensor in recognizing WNV in these cell types [17]. To date, no experiments have been published on the direct role of TLR7 and TLR8 in the priming of IFN responses after WNV infection in cells. A recent study demonstrated no decrease in systemic production of type I IFN in TLR7^−/−^ mice infected with WNV [18]. However, investigations with other RNA viruses suggest that the TLR7/MyD88/IRF-7 axis regulates type I IFN responses in specific subsets of dendritic cells [19],[20].

Studies with genetically deficient mice have established an important role of IRF-3 in protection against lethal WNV infection by controlling viral burden in peripheral and central nervous system (CNS) tissues without affecting the systemic type I IFN response [21]. Experiments performed with primary cells established that IRF-3 restricts WNV replication in cortical neurons and MEF through type I IFN-dependent mechanisms whereas in myeloid cells IRF-3 limits WNV infection independently of IFN by regulating basal or WNV-induced expression of host defense molecules [21]. More recent studies demonstrated a pivotal role of IRF-7 in vivo in controlling WNV infection [22]. IRF-7^−/−^ mice developed markedly elevated WNV burdens in multiple tissues and had a blunted systemic type I IFN response. Studies with primary cells showed that IRF-7 controls WNV infection largely through an IFN-α-dependent mechanism. Surprisingly, the IFN-β response remained intact suggesting possible redundant effects of IRF-3 and IRF-7 in regulating IFN-β gene expression. These results agree with an independent study in which IRF-7^−/−^ MEF infected with herpes simplex (HSV), vesicular stomatitis (VSV), or encephalomyocarditis (EMCV) virus showed residual IFN-β responses that were abolished in IRF-3^−/−^× IRF-7^−/−^ MEF [14].

To understand the combined roles of IRF-3 and IRF-7 in innate immune programs following WNV infection in vivo, we generated IRF-3^−/−^× IRF-7^−/−^ double knockout (DKO) mice. An absence of both IRF-3 and IRF-7 led to uncontrolled WNV replication in tissues and more rapid death than either of the single gene deletions. Although severe, the DKO phenotype did not recapitulate that observed in congenic IFN-αβR^−/−^ mice. Despite a complete absence of both IRF-3 and IRF-7, IFN-β induction remained largely intact in some cell types. Remarkably, in experiments with myeloid dendritic cells (mDC) ex vivo, the IFN-β response after WNV infection was abolished by the absence of IPS-1 but was largely unaffected by a combined deficiency or the transcription factors IRF-3 and IRF-7 or individual deficiencies of IRF-1, IRF-5, or IRF-8. However, pharmacological inhibition of signaling by both NF-κB and ATF-2/c-Jun, the two other factors, beside IRF-3 and IRF-7, that form the IFN-β gene transcriptional complex enhanceosome [23]–[25], strongly reduced the IFN-β response in DKO but not wild type mDC after WNV infection. These studies define cell type-specific molecular mechanisms and roles for IRFs in antiviral defense, and reveal a differential requirement for components of the enhanceosome in inducing the IFN-β gene in response to RNA viruses.

## Results

### Increased lethality of DKO mice after infection with WNV

**Figure 1:**
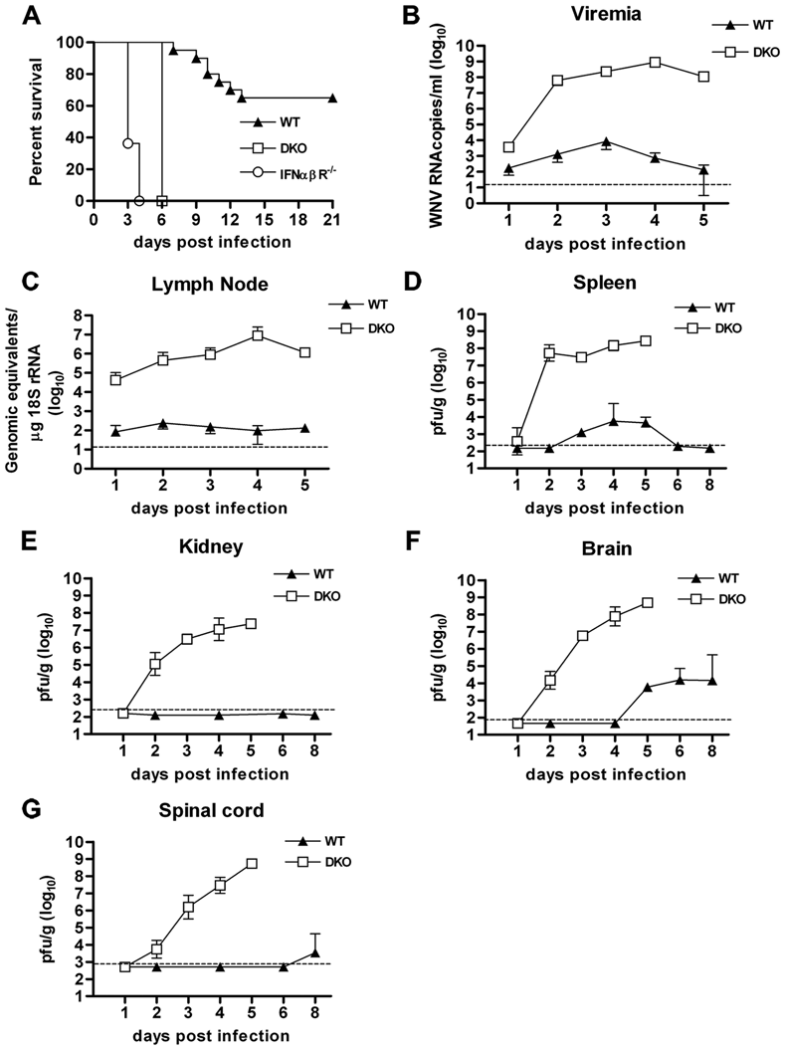
Survival and viral burden analysis in mice. A. Eight to 12 week-old C57BL/6 mice were inoculated with 10^2^ PFU of WNV by footpad injection and followed for mortality for 21 days. Survival differences were statistically significant between immunodeficient and wild type mice (n = 11, IFN-αβR^−/−^; n = 20, DKO; and n = 20, wild type mice, P<0.0001). Average survival time between IFN-αβR^−/−^ (3.5 days) and DKO (6 days) mice was also statistically different (P<0.001). B–G. Viral burden in peripheral and CNS tissues after WNV infection. WNV RNA in (B) serum and (C) draining lymph node, and infectious virus in (D) spleen, (E) kidney, (F) brain and (G) spinal cord were determined from samples harvested on the indicated days using qRT-PCR (B and C) or viral plaque assay (D–G). Data is shown as viral RNA equivalents or PFU per gram of tissue for 10 to 12 mice per time point. For all viral load data, the solid line represents the median PFU per gram at the indicated time point, and the dotted line represents the limit of sensitivity of the assay.

Because of the possible functional redundancy of IRF-3 and IRF-7, and to fully evaluate their net contribution to the regulation of the IFN-β transcriptional response after WNV infection, we generated IRF-3^−/−^× IRF-7^−/−^ (DKO) mice. These animals were infected subcutaneously with 10^2^ PFU of a highly pathogenic New York strain of WNV. Whereas wild type C57BL/6 mice exhibited a ∼60% survival rate, congenic DKO mice displayed a severe phenotype with 100% mortality and a mean time to death of 6.0 days (**Fig 1A**). In comparison, IRF-3^−/−^ or IRF-7^−/−^ single knockout mice infected with the same dose of WNV also had a 100% mortality rate but with mean time to deaths of 9.3 and 7.4 days, respectively [21],[22].

As IRF-3 and IRF-7 are key regulators of the type I IFN response to viral infection, we also compared the DKO phenotype with congenic IFN-αβR^−/−^ mice. Similar to what we previously observed [13],[26], IFN-αβR^−/−^ mice were vulnerable to WNV infection with 100% mortality by 4 days and a mean time to death of 3.5 days (**Fig 1A**, P<0.001 of average survival time compared to DKO mice). Thus, although a combined deficiency of IRF-3 and IRF-7 has a more severe phenotype after WNV infection than their respective single deficiencies, the survival pattern did not fully recapitulate that observed in IFN-αβR^−/−^ mice, suggesting type I IFN induction and/or regulation after WNV infection may require additional transcriptional regulators.

### DKO mice show uncontrolled WNV replication in tissues

To more completely evaluate the impact of the IRF-3 and IRF-7 deficiency on WNV pathogenesis in vivo, wild type and DKO mice were infected subcutaneously with 10^2^ PFU of WNV and viral burden was measured by fluorogenic quantitative RT-PCR or viral plaque assay at days 1, 2, 3, 4, 5, 6 and 8 in blood, peripheral organs (draining lymph nodes, spleen and kidney) and the CNS tissues (brain and spinal cord) (**Fig 1B–G**).

Elevated viremia was observed in DKO mice when compared to wild type mice (**Fig 1B**). By one day after infection, ∼10^1.5^-fold higher levels of viral RNA (P<0.0001) were detected in the DKO mice. Markedly enhanced (10^4.6^ to 10^6.2^-fold increase, P<0.0001) WNV RNA levels were observed in serum of DKO mice on days 2 through 5, after which most animals succumbed to infection.

In the draining lymph nodes, similarly high levels (10^2.8^ to 10^5.0^-fold increase, P<0.0001) of WNV RNA were observed in the DKO mice throughout infection (**Fig 1C**). In the spleen, infectious WNV was not detected in wild type mice until day 3. In contrast, all DKO mice (10 of 10) had markedly elevated viral titers by day 2 (mean titer of 10^7.7^ PFU/g) (**Fig 1D**). Although WNV infection gradually increased in the spleens of wild type mice at days 3 through 5, significantly higher (10^4.5^ to 10^4.9^-fold) viral burdens were observed in DKO mice. Altered tissue tropism was also observed in DKO mice with significant infection of the kidneys (e.g. 10^5^ PFU/g, by day 2 and 10^7.5^ PFU/g, by day 5, **Fig 1E**), whereas wild type mice showed no productive infection of the kidneys. Thus, a combined deficiency of IRF-3 and IRF-7 results in sustained and elevated WNV infection in peripheral compartments including spread to and propagation within normally non-permissive organs.

Analysis of viral burden in the brain and spinal cord showed a rapid entry of WNV into the CNS of DKO mice. WNV was detected in all brains from DKO mice at day 2 compared to wild type mice, where WNV was not observed until day 5 (**Fig 1F**). Notably, at day 5, DKO mice had ∼10^5.2^ -fold higher viral titers in the brain compared to wild type mice. A similar pattern was observed in the spinal cord of DKO mice with all animals showing markedly elevated viral loads after day 2 (**Fig 1G**). In contrast, in wild type mice, infectious WNV was not detected in the majority of animals until day 8 after infection. Thus, a combined absence of IRF-3 and IRF-7 results in early neuroinvasion and uncontrolled WNV replication.

### The systemic type I IFN response in DKO mice is blunted but not abolished

**Figure 2:**
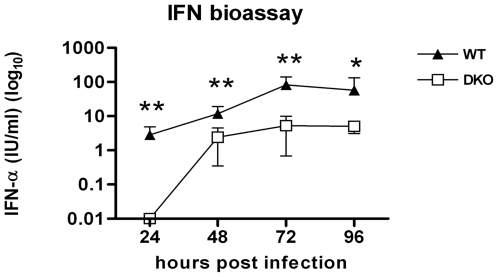
Levels of type I IFN levels in serum of wild type and DKO mice infected with WNV. Mice were inoculated with 10^2^ PFU of WNV by footpad injection and sacrificed at the indicated times. Type I IFN levels were determined from serum collected on days 1 to 4 after WNV infection by an EMCV bioassay in L929 cells. Data reflect averages of serum samples from 5 to 10 mice per time point and the data are expressed as international units (IU) of IFN-α per ml. The specificity of the assay was confirmed with an anti-IFN-αβR neutralizing antibody (data not shown). Asterisks indicate values that are statistically significant (**, P<0.005, *, P<0.05).

Previous studies have reported that an absence of IRF-3 in vivo does not profoundly reduce the levels of type I IFN in serum after WNV [21],[27] or other viral infections [14]. In contrast, IRF-7^−/−^ mice infected with WNV had a reduced but not abrogated systemic IFN response [22]. We therefore used the DKO mice to define whether signaling through IRF-3 could explain the residual systemic type I IFN response after WNV infection in IRF-7^−/−^ mice. Using a highly sensitive L929 cell bioassay, we compared the serum IFN levels in DKO and wild type mice after WNV infection. As observed previously [21],[22], in wild type mice type I IFN was detected in serum by day 1 with peak levels measured at days 3 and 4 after infection (**Fig 2**). In contrast, in the DKO mice, serum IFN levels at day 1 were below the detection level (0.01 IU/ml) of the assay. Despite the higher viremia, serum IFN levels in the DKO mice were reduced at days 2, 3 and 4 when compared to wild type animals (∼5 to 14-fold lower, P<0.005), but were not abolished. This antiviral activity in the serum of infected DKO mice was confirmed as type I IFN-dependent by depletion experiments with a neutralizing mAb against the IFN-α/βR (data not shown). Thus, a combined deficiency of IRF-3 and IRF-7 in vivo delays and diminishes the accumulation of type I IFN in serum. However, over time, type I IFN accumulates in the serum of DKO mice after WNV infection. Thus, additional regulatory factors must contribute to the systemic IFN response. Notably, these results agree with a recent study in which independently-generated DKO mice infected with mouse cytomegalovirus (MCMV) showed a residual systemic IFN response that was entirely specific for IFN-β [28].

### IRF-3 and IRF-7 are required for the type I IFN and ISG response in MEF after WNV infection

**Figure 3:**
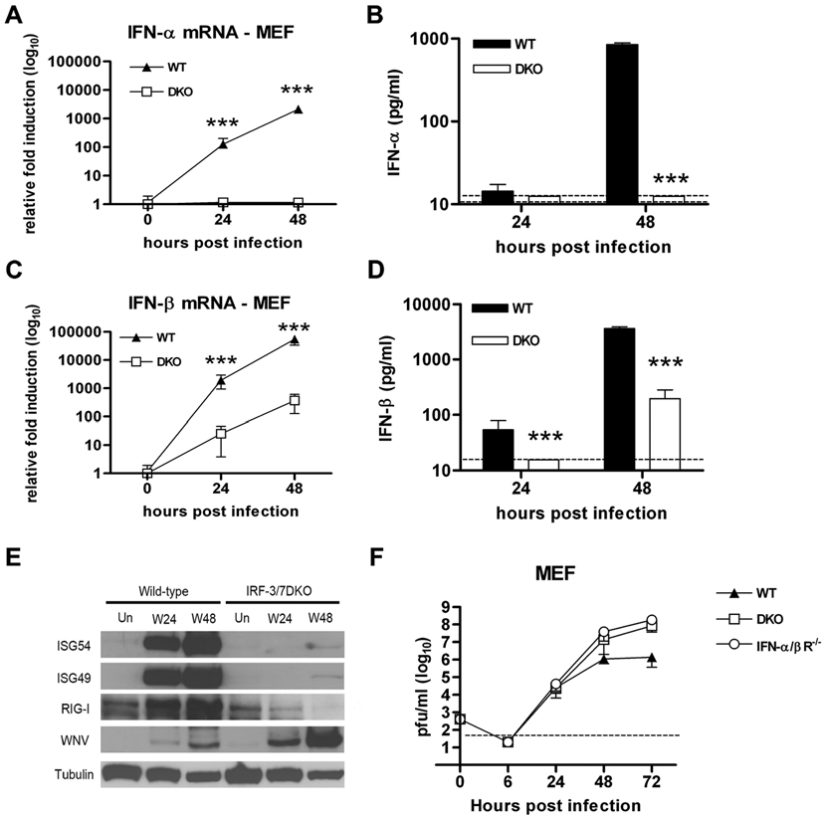
IRF-3 and IRF-7 control the IFN-α/β gene induction and ISG expression in MEF. A–D. MEF generated from wild type or DKO mice were infected at an MOI of 0.1 and analyzed for IFN-α/β gene induction. Total RNA from uninfected and WNV-infected MEF was harvested at the indicated times after infection and levels of (A) IFN-α and (C) IFN-β mRNA were measured by qRT-PCR. Data are normalized to 18S rRNA and are expressed as the relative fold increase over normalized RNA from uninfected controls. Accumulation of (B) IFN-α and (D) IFN-β protein in supernatants was evaluated by ELISA. E. Whole cell lysates were generated at the indicated times from wild type or DKO MEF that were uninfected (Un) or infected with WNV (W). Protein levels of ISG49, ISG54, RIG-I, WNV and tubulin were examined by immunoblot analysis. F. MEF generated from wild type, IFN-αβR^−/−^ and DKO mice were infected at an MOI of 0.001 and virus production was evaluated by plaque assay. The data is the average of at least three independent experiments performed in quadruplicate, (***, P<0.0001).

To better understand the net effect of IRF-3 and IRF-7 on WNV infection and induction of a protective IFN response, we infected primary cells from DKO and wild type mice. Because MEF have been studied extensively in virus infection-host immune response assays [16],[29],[30], we initially evaluated the effect of an IRF-3 × IRF-7 deficiency in these cells. Previous experiments had shown a blunted IFN-α response and a normal IFN-β response in IRF-7^−/−^ MEF infected with WNV [22]. In contrast, IRF-3^−/−^ MEF had a diminished IFN-α and -β response early after infection but developed normal levels at later time points (S. Daffis and M. Diamond, unpublished data). In DKO MEF, IFN-α mRNA and protein secretion were completely abolished after WNV infection (**Fig 3A and 3B**). Similarly, levels of IFN-β mRNA were strongly reduced at 24 h (∼70-fold decrease, P<0.0001) and 48 h after infection (∼130-fold decrease, P<0.0001) but not entirely abolished (**Fig 3C**). Measurement of secreted IFN-β in the cell supernatants corroborated these findings as a ∼4 and 20-fold reduction (P<0.0001) was observed in DKO MEF at 24 and 48 hours, respectively (**Fig 3D**). Thus, normal induction of IFN-α and IFN-β in MEF after WNV infection primarily requires transcriptional activation by IRF-3 and IRF-7.

We next assessed the expression pattern of selected ISG including ISG49, ISG54 and RIG-I by Western blot analysis. In wild type MEF, these proteins become rapidly induced at 24 h after infection and were sustained at 48 h. In contrast, induction was absent in DKO MEF at 24 h after infection and levels were comparably reduced at 48 h (**Fig 3E**). To assess how this phenotype affected WNV replication, multi-step viral growth analysis was performed (**Fig 3F**). Although no difference in viral titers was observed at 24 hours, enhanced WNV replication was seen at 48 h (13-fold, P = 0.004) and 72 h (58-fold, P<0.0001) in DKO MEF. Moreover, the levels of WNV at 48 and 72 h in the DKO MEF were greater than those previously observed with the single IRF-3^−/−^ or IRF-7^−/−^ MEF (S. Daffis and M. Diamond, unpublished results and [22]). As IRF-3 and IRF-7 appear to primarily regulate the IFN and ISG responses in MEF, we predicted that the WNV replication phenotype in the DKO MEF should not differ substantially from congenic IFN-αβR^−/−^ MEF. Indeed, when directly compared, only small differences in viral growth were observed between DKO and IFN-αβR^−/−^ MEF (2.7 fold, P<0.03 at 48 h and 2.3 fold, P<0.002 at 72 h) (**Fig 3F**).

### IRF-3 and IRF-7 regulate the type I IFN and ISG response in cortical neurons after WNV infection

**Figure 4:**
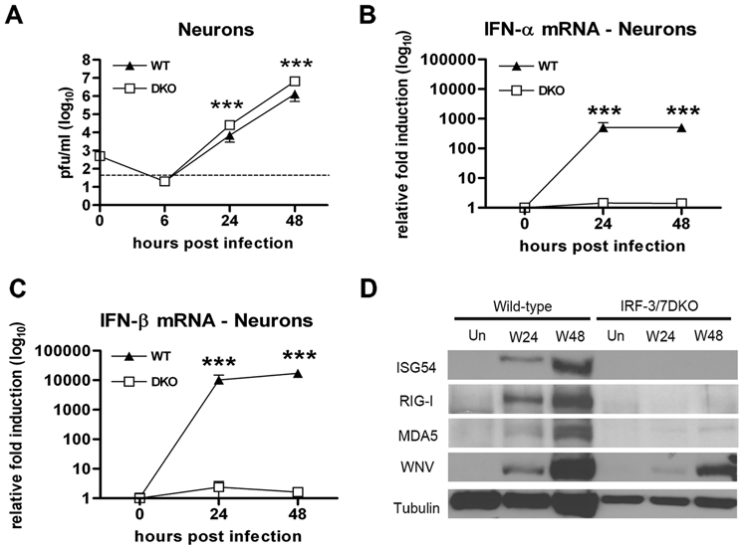
IRF-3 and IRF-7 restrict WNV infection by regulating the IFN-α/β response and ISG expression in primary cortical neurons. A. Primary cortical neurons generated from wild type or DKO mice were infected at an MOI of 0.001 and virus production was evaluated at the indicated times by plaque assay. Values are an average of triplicate samples generated from three independent experiments. Asterisks indicate values that are statistically significant (***, P<0.0001). B and C. Levels of (B) IFN-α and (C) IFN-β mRNA in WNV-infected cortical neurons were measured by qRT-PCR as described in the legend of Figure 3. D. Whole cell lysates were generated at the indicated times from wild type or DKO MEF that were uninfected (Un) or infected with WNV (W). Protein levels of ISG54, RIG-I, and MDA5 were examined by immunoblot analysis. The data is the average of at least three independent experiments performed in quadruplicate (***, P<0.0001).

To evaluate the combined roles of IRF-3 and IRF-7 in control of viral replication and the IFN response in neuronal cells, we assessed WNV infection of primary cortical neurons isolated from DKO mice. Analysis of viral growth kinetics confirmed that IRF-3 and IRF-7 restrict WNV replication as a ∼5 to 6-fold increase (P<0.0001) in viral titer was observed at 24 h and 48 h compared to wild type cells (**Fig 4A**). Somewhat surprisingly, the DKO neurons did not show increased replication relative to cells lacking either IRF-3 or IRF-7 (data not shown and [21],[22]). The relatively modest replication phenotype in the absence of IRF-3 and IRF-7 is consistent with only a small IFN-dependent antiviral effect in these cells: IFN-α or -β pre-treatment inhibits WNV infection in cortical neurons a maximum of 5 to 8-fold [13]. Analysis of the IFN response of WNV-infected DKO neurons showed a complete ablation of the IFN-α gene induction confirming results with the IRF-7^−/−^ cortical neurons [22] (**Fig 4B**). In contrast, and unlike that observed with MEF or other primary myeloid cells (see below), induction of IFN-β mRNA in DKO cortical neurons was also entirely abolished (**Fig 4C**). Consistent with this, analysis of ISG in DKO neurons showed a complete loss of induction of ISG54, RIG-I and MDA5 following WNV infection (**Fig 4D**). Thus, in cortical neurons coordinate signals through the transcriptional regulators IRF-3 and IRF-7 are required for IFN-α and IFN-β gene induction after WNV infection.

### IFN-β response in Mφ after WNV infection is partially IRF-3 and IRF-7-dependent

**Figure 5:**
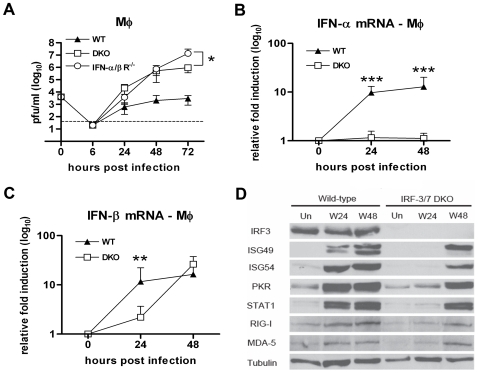
IRF-3 and IRF-7 partially modulate the IFN-β response and ISG expression in primary Mφ. A. Mφ generated from wild type, IFN-αβR^−/−^ and DKO mice were infected at an MOI of 0.01 and virus production was evaluated at the indicated times post infection by plaque assay. Values are an average of quadruplicate samples generated from at least three independent experiments. B. Whole cell lysates were generated at the indicated times from wild type and DKO Mφ that were uninfected (Un) or infected with WNV (W). Protein levels of ISG49, ISG54, PKR, STAT1, RIG-I, MDA5 and tubulin were examined by immunoblot analysis. C and D. The induction of (C) IFN-α and (D) IFN-β mRNA in WNV-infected Mφ was analyzed by qRT-PCR as described in Figure 3. Asterisks indicate values that are statistically significant (***, P<0.0001, **, P<0.005, *, P<0.05).

As previous studies suggested cell type-specific differences in type I IFN induction [21], we evaluated the effect of the combined IRF-3 and IRF-7 deficiency in macrophages (Mφ), a cell type that is permissive to WNV in vivo [26]. Prior experiments established that Mφ lacking either IRF-3 or IRF-7 were more susceptible to WNV infection [21],[22]. Analogously, DKO Mφ supported increased WNV replication (∼35, 250 and 310-fold, P<0.0001 at 24, 48 and 72 h after infection, respectively) compared to wild type or even IRF-3^−/−^ or IRF-7^−/−^ singly deficient cells (**Fig 5A** and [21],[22]). In contrast to that observed with MEF (see **Fig 3**), the DKO Mφ produced ∼14-fold less (P<0.05) WNV at 72 h compared to IFN-αβR^−/−^ Mφ infected in parallel. Thus, an absence of both IRF-3 and IRF-7 in Mφ did not recapitulate the replication phenotype of IFN-αβR^−/−^ cells. As such a discrepancy could be related to differences in the type I IFN and/or ISG response, we analyzed these in DKO Mφ after WNV infection. As expected, levels of IFN-α mRNA were completely abolished (**Fig 5B**), consistent with results from IRF-7^−/−^ Mφ [22]. Whereas IFN-β mRNA levels were reduced at 24 h after infection in DKO Mφ, they accumulated to normal levels by 48 h (**Fig 5C**). Western blot analysis of ISG expression confirmed this pattern, as the early induction of several ISG was altered at 24 h but not 48 h following WNV infection (**Fig 5D**). Thus, after WNV infection of Mφ, IRF-3 and IRF-7 coordinately regulate the early but are dispensable for the later IFN-β and ISG responses.

### IRF-3 and IRF-7 are also dispensable for the IFN-β response in mDC after WNV infection

**Figure 6:**
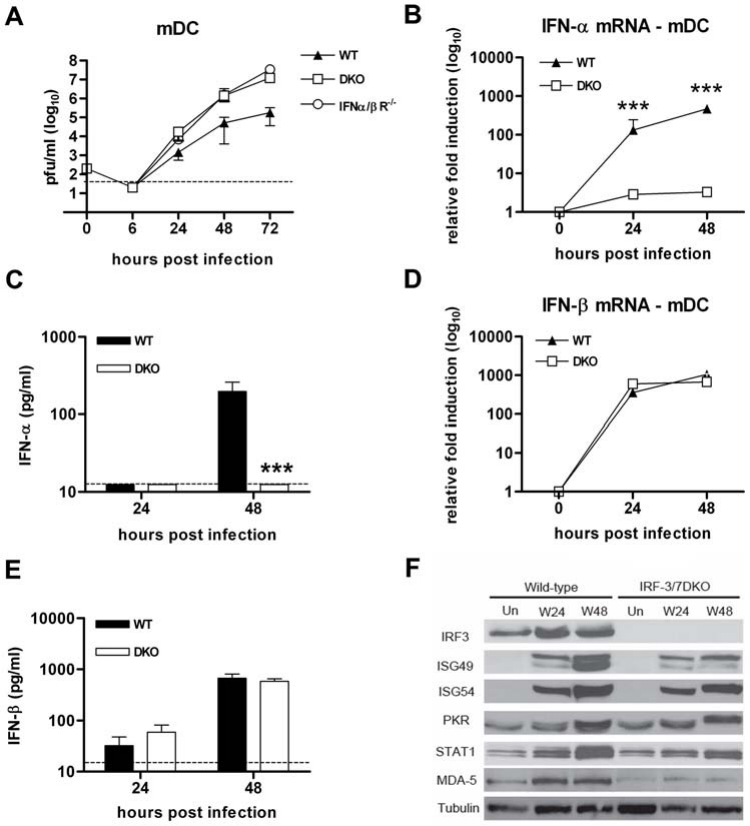
The WNV-induced IFN-β response and ISG expression is primarily IRF-3 and IRF-7-independent in mDC. A. mDC generated from wild type, IFN-αβR^−/−^ and DKO mice were infected at an MOI of 0.001 and virus production was evaluated at the indicated times post infection by plaque assay. Values are an average of quadruplicate samples generated from at least three independent experiments (***, P<0.0001). B–E. Levels of (B) IFN-α and (D) IFN-β mRNA as well as (C) IFN-α and (E) IFN-β protein in WNV-infected mDC were measured by qRT-PCR or ELISA as described in the legend of Figure 3. F. Whole cell lysates were generated at the indicated times from wild type and DKO mDC that were uninfected (Un) or infected with WNV (W). Protein levels of ISG49, ISG54, PKR, STAT1, RIG-I, MDA5 and tubulin were examined by immunoblot analysis.

Because mDC are likely early targets for WNV infection in animals [31]–[33] and help orchestrate innate and adaptive antiviral immune responses [34], we evaluated IFN and ISG responses in bone marrow-derived mDC. Prior studies showed that mDC lacking either IRF-3 or IRF-7 support enhanced WNV replication, IRF-3^−/−^ mDC developed normal IFN-α/β responses after WNV infection, and IRF-7^−/−^ mDC had reduced IFN-α but relatively intact IFN-β responses after WNV infection (S. Daffis and M. Diamond, unpublished results and [22]). Multi-step growth curve analysis of DKO mDC infected with WNV showed a higher viral burden compared to wild type cells (16 to 95-fold, P<0.0001) and IRF-3^−/−^ or IRF-7^−/−^ mDC (**Fig 6A**, S. Daffis and M. Diamond, unpublished results, and [22]). The viral titers in DKO mDC were similar to those obtained in IFN-αβR^−/−^ mDC, suggesting a defect in type I IFN signaling in the DKO cells. Surprisingly, whereas levels of IFN-α mRNA and protein were abolished (**Fig 6B and 6C**), induction of IFN-β gene and protein production was not significantly affected (P>0.2) after WNV infection of DKO mDC (**Fig 6D and 6E**). Thus, in mDC, IRF-3 and IRF-7 regulate the IFN-α response but are largely dispensable for inducing IFN-β after WNV infection. Western blot analysis of ISG corroborated these findings as similar levels of ISG were observed in wild type and DKO mDC at 24 and 48 h after WNV infection (**Fig 6F**). These data suggest that the expression of ISG is primarily IFN-β-dependent or that the IFN-α and -β have redundant effects in these cells. Nonetheless, as higher viral replication was sustained in DKO mDC despite a relatively normal IFN-β response and ISG expression profile, it remains possible that IRF-3 directly regulates expression of a key subset of ISG that accounts for anti-WNV activity in this cell type.

**Figure 7:**
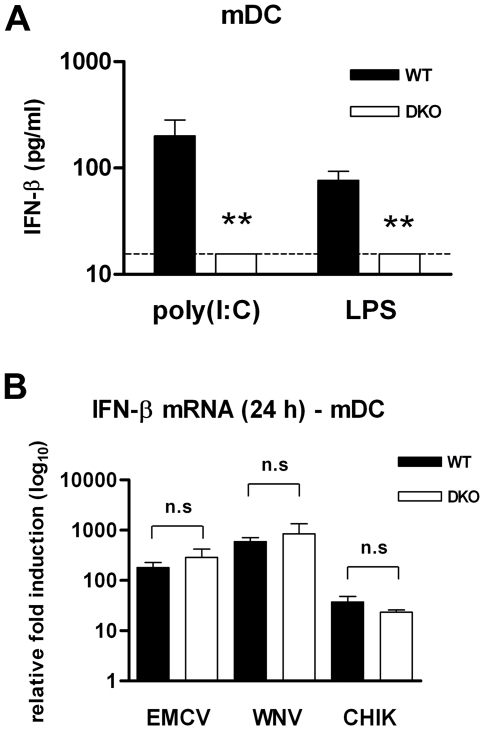
The IFN-β response in mDC after TLR stimulation and viral infection. mDC generated from wild type and DKO mice were (A) stimulated with 50 µg/ml of poly(I∶C) or 2 µg/ml LPS for 24 h or (B) infected with EMCV (MOI 0.1), WNV (MOI 0.1) and CHIK (MOI 1) for 24 h. Levels of IFN-β were measured either by ELISA or qRT-PCR. Values are an average of triplicate samples generated from three independent experiments. Asterisks indicate values that are statistically significant (***, P<0.0001, ** P<0.0005) from wild type cells; n.s. indicates differences that were not statistically significant.

To determine whether the IFN-β induction response in DKO mDC was specific for WNV, we performed experiments with agonists for the TLR3 and TLR4 pathways and with unrelated viruses (Chikungunya virus (CHIK), an emerging human alphavirus, and EMCV, a model rodent picornavirus). As expected, wild type mDC that were treated extracellularly with TLR3 (poly I∶C) or TLR4 (LPS) agonists rapidly induced IFN-β mRNA (**Fig 7A**). In DKO cells, and in contrast to that observed with WNV, the IFN-β response downstream of TLR treatment was effectively abolished. Thus, activation of the IFN-β response in mDC after TLR3 and TLR4 stimulation requires both IRF-3 and IRF-7. To define whether the IRF-3/IRF-7-independent IFN-β response was specific to WNV, we infected mDC with additional RNA viruses (**Fig 7B**). In wild type mDC, EMCV infection induced a robust IFN-β response similar to WNV, and high levels of IFN-β mRNA were detected by 24 hours post infection (∼100-fold increase). Infection with CHIK at a higher MOI also resulted in a similar IFN-β gene induction at 24 hours post infection. In DKO cells, the IFN-β response after infection with WNV, EMCV, and CHIK was equivalent to that observed in wild type cells. Thus, in mDC, IRF-3 and IRF-7 are dispensable for activation of IFN-β gene transcription, not only in response to WNV but also to other positive strand RNA viruses that are genetically unrelated.

### The IFN-β response in mDC after WNV infection does not rely on type I IFN signaling and IRF-1 or IRF-8

**Figure 8:**
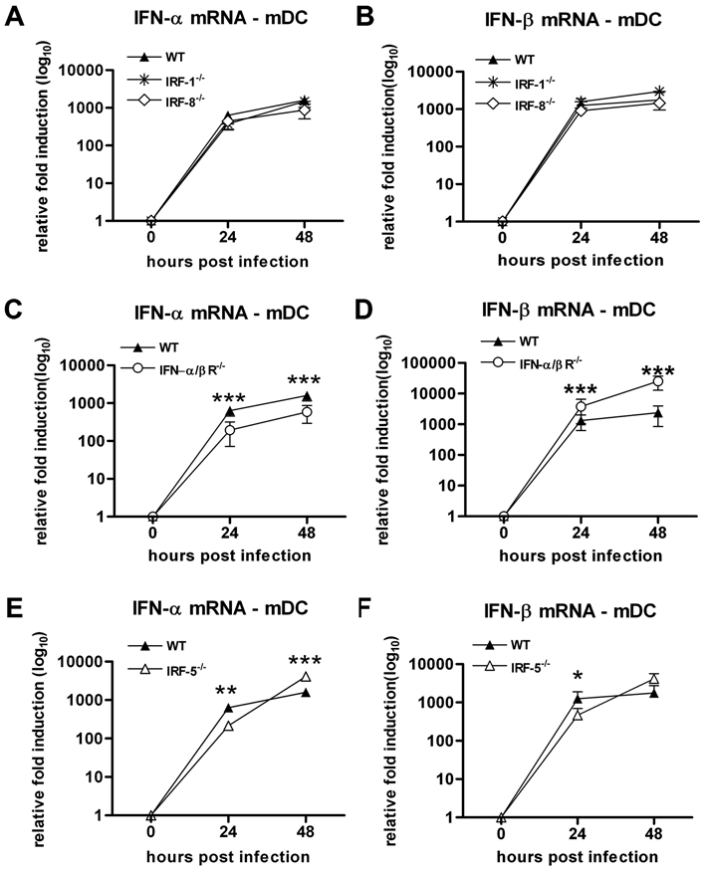
The IFN-β response in mDC is IRF-1, IRF-8, IFN-α/β-independent but partially IRF-5-dependent. mDC generated from wild type, (A and B) IRF-1^−/−^, IRF-8^−/−^, (C and D) IFN-α/βR^−/−^, or (E and F) IRF-5^−/−^ mice were infected at an MOI of 0.1 and levels of IFN-α (A, C, and E) and IFN-β (B, D, and F) mRNA were measured by qRT-PCR. Values are an average of triplicate samples generated from three independent experiments. Asterisks indicate values that are statistically significant (***, P<0.0001, **, P<0.005, *, P<0.05) compared to wild type.

To begin to investigate the mechanism(s) that regulate IFN-β gene induction in mDC, we infected IRF-1^−/−^ and IRF-8^−/−^ mDC with WNV. These transcription factors have been implicated in type I IFN gene transcription in DC after TLR stimulation or viral infection [35],[36]. Notably, no difference in IFN-α and IFN-β mRNA levels was observed in IRF-1^−/−^ mDC (**Fig 8A and 8B**). Subsequently, we assessed whether the regulator of IFN-β transcription was IFN-inducible, as has been suggested for IRF-8 in the context of infection by MCMV [36] or paramyxovirus [37]. IFN-αβR^−/−^ mDC were infected with WNV and the levels of IFN-α and IFN-β mRNA were measured. Whereas the IFN-α response was diminished in these cells, likely because of the abolition of the type I IFN positive feedback loop (**Fig 8C**), the IFN-β gene response in IFN-αβR^−/−^ mDC was greater (5 to 10-fold, P<0.05) compared to wild type cells (**Fig 8D**). These results suggest that, unlike the regulation of the IFN-α response, activation of the IFN-β gene after WNV infection in mDC is independent of the type I IFN positive feedback loop. Consistent with this, no difference in IFN-α and IFN-β induction was observed after WNV infection of IRF-8^−/−^ mDC (**Fig 8A and 8B**).

Recent studies have suggested that IRF-5 can activate transcription of IFN and other inflammatory cytokine genes after viral infection or TLR stimulation [38]–[41]. As IRF-5 also has been implicated in IFN production and protection in vivo after infection by negative strand RNA viruses (Newcastle Disease virus (NDV) and VSV) and DNA viruses (HSV) [38],[42], we evaluated whether the transcriptional signal after WNV infection of mDC cells was dependent on IRF-5. IRF-5^−/−^ mDC showed only a modest yet reproducible reduction (2.5-fold, P<0.05) in IFN-β gene transcription within 24 hours of WNV infection (**Fig 8D**). By 48 hours, however, these deficits were no longer apparent as equivalent or even greater levels of IFN-β mRNA were detected in IRF-5^−/−^ cells. Taken together, our experiments suggest that IRF-1 and IRF-8 are dispensable for induction of IFN-β gene transcription in mDC, whereas an IRF-5 has a small and transient regulatory effect in these cells after WNV infection.

### A combined inhibition of NF-κB and ATF-2/c-Jun in DKO mDC reduces the IFN-β response after WNV infection

**Figure 9:**
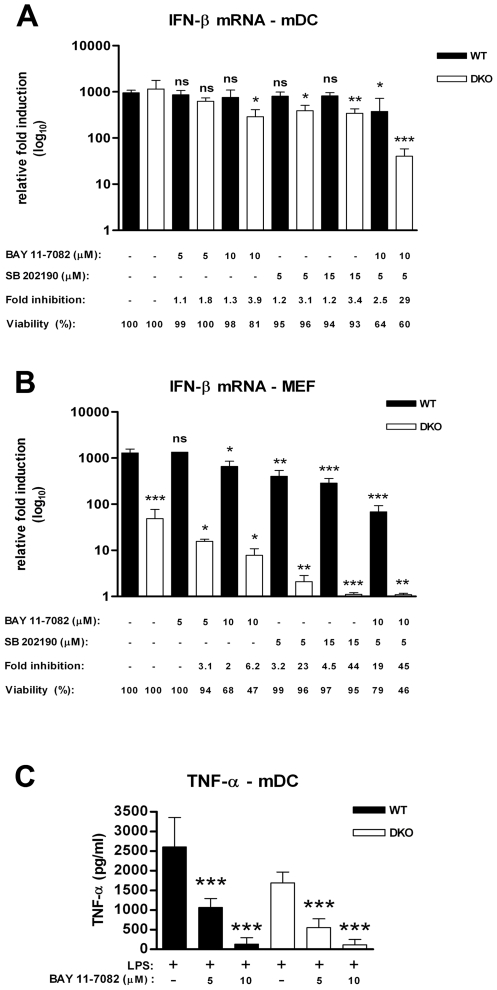
Pharmacological inhibition of NF-κB and p38 in wild type and DKO mDC and MEF. A–B. mDC (A) and MEF (B) generated from wild type and DKO mice were infected at an MOI of 0.1 in the presence of 1% DMSO or increasing concentrations of BAY 11-7082 (in 1% DMSO) and/or increasing concentrations of SB 202190 (in 1% DMSO). Levels of IFN-β mRNA were evaluated at the indicated times post-infection by qRT-PCR. Cell viability was analyzed using a cytotoxicity assay as described in the Materials and Methods. C. Efficiency of BAY 11-7082 in inhibiting NF-κB transcriptional activity. Wild type and DKO mDC were treated with 1% DMSO or 5 µM and 10 µM of BAY 11-7082 and stimulated with LPS (2 µg/ml) for 24 hours. Levels of secreted TNF-α were measured by ELISA. Values are an average of duplicate samples generated from three independent experiments. Asterisks indicate values that are statistically significant (***, P<0.0001, **, P<0.005, *, P<0.05); n.s. indicates differences that were not statistically significant.

The transcriptional activation of the IFN-β gene requires full occupancy of an enhancer complex known as the “enhanceosome” [23]–[25]. In vitro, the active IFN-β enhanceosome is formed after association of the transcription factors IRF-3, IRF-7, ATF-2/c-Jun, and NF-κB. Although synergy in transcription of the IFN-β gene occurs after coordinate binding of these regulatory factors in several cell types [25], the mechanistic basis for full occupancy of the enhanceosome remains incompletely understood. Since a combined deficiency of IRF-3 and IRF-7 only modestly reduced the IFN-β gene induction in mDC after WNV infection, we hypothesized that the residual IFN-β activity could occur through a NF-κB-dependent and/or ATF-2/c-Jun-dependent signal that is mediated directly through PRR signaling. To test this possibility, we used the highly specific and validated pharmacological inhibitors of NF-κB (BAY 11-7082) [43],[44] and p38 MAP kinase (SB 202190), which is essential for phosphorylating and activating the ATF-2/c-Jun complex [45]. Treatment of DKO mDC with increasing concentrations of BAY 11-7082, the NF-κB inhibitor, yielded only a small reduction of WNV-induced IFN-β response (maximum of 3.9 fold decrease, P = 0.006) (**Fig 9A**). In contrast, the LPS-induced TNF-α response, which is dominantly regulated by NF-κB [46],[47], was dose-dependently inhibited in wild type and DKO mDC treated with BAY 11-7082 (**Fig 9C**). Analogously, treatment of DKO mDC with SB 202190 had a limited effect on the WNV-induced IFN-β response (3.5 fold decrease, P = 0.02) (**Fig 9A**). Thus, in mDC lacking IRF-3 and IRF-7, inhibition of NF-κB or ATF-2/c-Jun alone only modestly reduced the IFN-β gene transcription after WNV infection. However, a combined inhibition of both NF-κB and ATF-2/c-Jun in DKO mDC strongly diminished the IFN-β response (29-fold decrease, P<0.0001). In contrast, a more subtle decrease (2.5-fold decrease, P = 0.006) of IFN-β transcription after WNV infection was observed in wild type mDC that retained expression of IRF-3 and IRF-7. Parallel experiments using an ATP metabolism assay confirmed that the difference in effect by the inhibitors was not due to differential cytotoxicity between wild type and DKO cells (**Fig 9A**). These experiments suggest that, in mDC, efficient activation of the IFN-β gene does not require the full enhanceosome occupancy, as a combined functional absence of IRF-3, IRF-7, NF-κB and ATF-2/c-Jun is necessary to strongly inhibit the IFN-β gene activation after WNV infection.

Since the activation of the IFN-β response in MEF was markedly reduced in the absence of both IRF-3 and IRF-7, we hypothesized that, in contrast to that observed in DC, the regulation of the IFN-β gene in MEF would depend more strongly on the full occupancy of the enhanceosome with canonical constituents. To assess this, wild type MEF were treated with BAY 11-7082 and/or SB 202190, infected with WNV, and levels of IFN-β mRNA were measured. In contrast to that observed with wild type mDC, inhibition of NF-κB in wild type MEF slightly decreased the IFN-β response (∼2-fold decrease, P = 0.01) (**Fig 9B**). Similarly, inhibition of ATF-2/c-Jun in wild type MEF also reduced the IFN-β response (∼5-fold decrease, P = 0.007). More strikingly, inhibition of both NF-κB and ATF-2/c-Jun in wild type MEF strongly attenuated the IFN-β gene transcription (∼20-fold decrease, P<0.0001). Thus, disruption of function of single components of the enhanceosome is sufficient to reduce the IFN-β transcriptional response in MEF. Consistent with this, inhibition of NF-κB and/or ATF-2/c-Jun in DKO MEF essentially abolished the residual IFN-β response (**Fig 9B**). Taken together, these data suggest that optimal regulation of the IFN-β gene transcription in mDC, in contrast to MEF, does not require complete occupancy four canonical components of the enhanceosome.

### A deficiency of IPS-1 abolishes the IFN-β response in MEF and mDC

**Figure 10:**
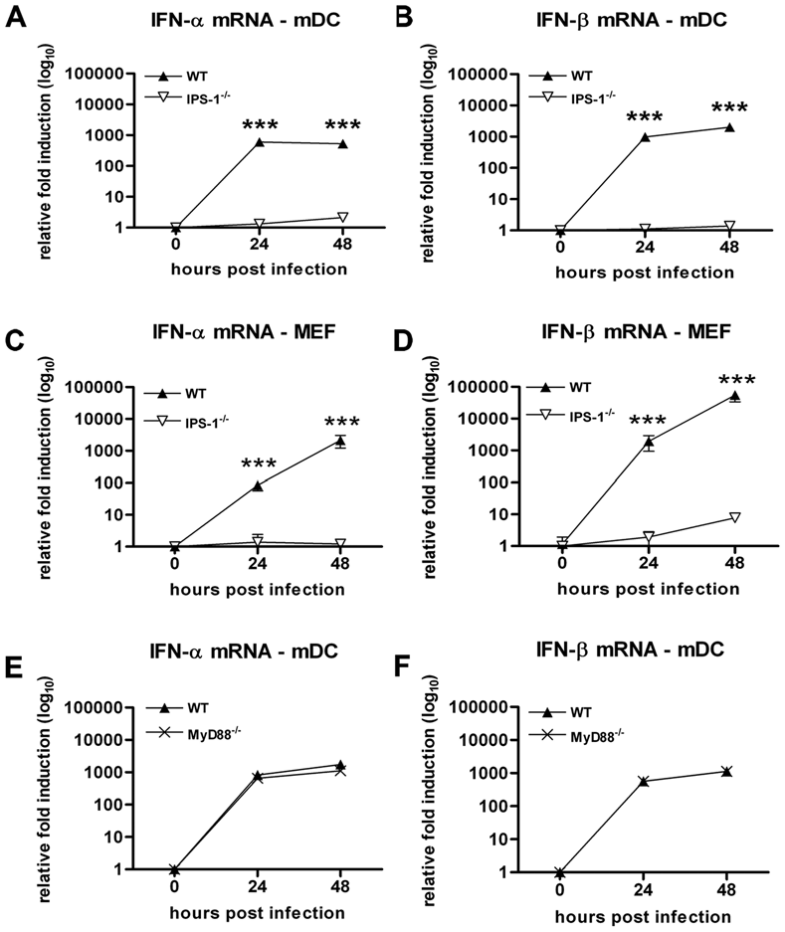
The IFN-β response in mDC and MEF is IPS-1-dependent but MyD88-independent. mDC and MEF generated from wild type, IPS-1^−/−^ and MyD88^−/−^, mice were infected at an MOI of 0.1 and levels of (A, C, and E) IFN-α and (B, D, and F) IFN-β mRNA were quantified by qRT-PCR. Values are an average of duplicate samples generated from three independent experiments. Asterisks indicate values that are statistically significant (***, P<0.0001).

Immune detection of WNV by mDC likely occurs through the cytosolic PRR, RIG-I and MDA5 and requisite signaling through the IPS-1 adaptor protein; this leads to activation of downstream IRF family proteins and IFN gene transcription [16]. To better understand the signaling pathway between PRR and IFN gene induction after exposure to WNV, we infected IPS-1^−/−^ MEF and mDC. Whereas levels of IFN-α and -β mRNA increase over time in wild type mDC and MEF, induction of both was virtually abolished in IPS-1^−/−^ cells (**Fig 10A–D**). Consistent with this, we did not detect any secreted IFN-α and β from IPS-1^−/−^ DC or MEF supernatants (data not shown). Thus, the induction of type I IFN genes in mDC or MEF after WNV infection is entirely dependent on IPS-1 signaling. Since IRF-5 may play a partial role in IFN-β gene regulation in mDC (**Fig 8D**) and since MyD88 has been suggested to mediate the TLR- and IRF-5-dependent cell type-specific induction of type I IFN after NDV infection [38], we also assessed the role of MyD88 in triggering the IFN-β response in mDC. As shown (**Fig 10E and F**), the IFN-α and β transcription induced by WNV infection in mDC was essentially independent of MyD88. Thus, an optimal IFN-β transcriptional signal in mDC after WNV infection is generated through a pathway that requires IPS-1, a subset of the four (IRF-3, IRF-7, NF-κB and ATF-2/c-Jun) components of the canonical enhanceosome and possibly, IRF-5, after a MyD88-independent activation signal.

### TRAF3 and TBK1 regulate the early IPS-1-dependent type I IFN response whereas TRAF6 modulates a later phase

**Figure 11:**
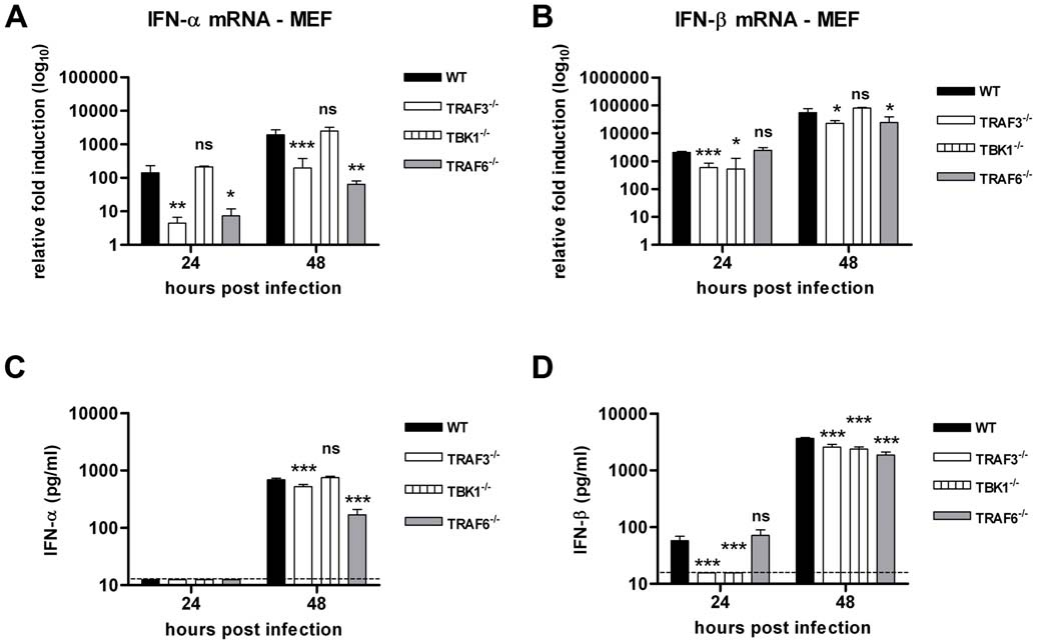
The early phase of the type I IFN regulation in MEF partially involves TRAF3 and TBK1 whereas the late phase requires TRAF6. TRAF3^−/−^, TBK1^−/−^ and TRAF6^−/−^ MEF were infected at an MOI of 0.1 and levels of (A and C) IFN-α and (B and D) IFN-β mRNA and secreted protein were measured by qRT-PCR and ELISA. Since basal mRNA expression of the IFN-β gene in uninfected TBK1^−/−^ and TRAF6^−/−^ MEF was lower than that observed in congenic wild type cells, for these cells only, we compared IFN-β mRNA levels to the wild type MEF. Values are an average of duplicate samples generated from three independent experiments. Asterisks indicate values that are statistically significant (***, P<0.0001, **, P<0.005, *, P<0.05); n.s. indicates differences that were not statistically significant.

Although our experiments suggested that IFN-β induction after WNV infection required IPS-1 yet was mediated by a cell type-specific complex of transcriptional regulators, the signaling adaptors that connected these pathways remained uncertain. As IPS-1-dependent regulation of the type I IFN genes in response to VSV and Sendai virus requires activation of TRAF3 [48], we infected TRAF3^−/−^ MEF with WNV and measured the levels of IFN-β mRNA and secreted cytokine. TRAF3^−/−^ MEF showed a significant reduction of IFN-β mRNA levels at 24 hours and 48 hours post infection (7.8-fold, P<0.0001 and 2.4-fold, P<0.05, respectively), which was confirmed by measuring IFN-β in the cell supernatant (3.7- and 1.4-fold decrease, respectively, P<0.0001) (**Fig 11B and 11D**). These data suggest that initial IPS-1-dependent trigger of the IFN-β response after WNV infection is mediated at least partially by TRAF3. Consistent with this, the levels of IFN-α mRNA and protein, which reflect the type I IFN positive feedback, also were decreased in TRAF3^−/−^ MEF (mRNA: 32-fold and 36- fold decreases, P<0.0001; protein: 1.3-fold, P<0.0001) (**Fig 11A and 11C**). As TBK1 is a kinase that reportedly mediates the signal to induce IFN production downstream of the IPS-1/TRAF3 axis [49], we assayed IFN-α/β induction after WNV infection in TBK1^−/−^ MEF. Similar to that observed in TRAF3^−/−^ MEF, the levels of mRNA and more importantly, secreted IFN-β were decreased in TBK1^−/−^ MEF (3.7- and 1.5-fold decrease, respectively, P<0.0001) (**Fig 11D**). However, levels of IFN-α mRNA and secreted cytokine in TBK1^−/−^ MEF were not different compared to wild type MEF (P≥0.2) (**Fig 11A and 11C**). Thus, TBK1 appears to mediate the early IFN-β response downstream of TRAF3 after WNV infection but appears dispensable for the later activation of IFN amplification loop. Collectively, these data suggest that, in MEF, the early IFN-β response depends on a signaling pathway involving IPS-1, TRAF3, and TBK1 whereas the late IFN-β response may also partially involve TRAF3. Unfortunately, because TRAF3^−/−^ and TBK1^−/−^ mice are lethal shortly after birth [50],[51], we could not confirm this signaling pathway in mDC.

Recent studies with VSV-infected cells have suggested that TRAF6 regulates the IPS-1-dependent signal to induce type I IFN [52]. To evaluate the contribution of TRAF6 to induction of the IFN response after WNV infection, levels of mRNA and IFN-β secreted protein were assayed in TRAF6^−/−^ MEF. In contrast to that observed in TRAF3^−/−^ MEF, levels of mRNA and secreted IFN-β in TRAF6^−/−^ cells supernatants were equivalent to wild type cells at 24 hours post infection (P>0.2) but slightly reduced at 48 hours (∼2-fold decrease, P<0.0001) (**Fig 11D**). TRAF6^−/−^ MEF, however, exhibited reduced IFN-α mRNA levels and protein secretion (240-fold and 4-fold reduction, P<0.0001) despite a relatively normal early IFN-β response (**Fig 11A and 11C**). Thus, TRAF6 appears dispensable for the early IFN-β gene response but required for amplifying the late IFN-α and -β responses in MEF. Our experiments suggest that TRAF3 and TRAF6 have distinct and complementary functions in activating the type I IFN response in MEF after WNV infection.

## Discussion

Early protection of the host against viral infections relies on the rapid detection of the pathogen and optimal activation of the type I IFN response. Here, using mice and primary cells lacking expression of both IRF-3 and IRF-7, we demonstrate that these transcriptional activators have essential non-redundant functions: DKO mice exhibited a more rapid lethality with enhanced tissue viral burden compared to mice lacking either IRF-3 or IRF-7. Associated with this profound virological phenotype, DKO mice had attenuated levels of systemic type I IFN. A combined deficiency of IRF-3 and IRF-7 however, showed heterogeneous cellular IFN-β but not -α responses. Whereas IFN-α was virtually abolished in all DKO cells tested, the IFN-β response was sustained in DKO Mφ and mDC. The uncoupling of virus induction of IFN-β from IFN-α in DKO Mφ and mDC establishes a cell-type specificity and IRF-3/IRF-7-independence of this pathway.

The combined absence of IRF-3 and IRF-7 largely abrogated the IFN-α and β responses in primary fibroblasts and cortical neurons. This data agrees with previous experiments showing loss of IFN-α and -β gene induction in DKO MEF infected with HSV-1, VSV, or EMCV [14]. In contrast, a deficiency of both IRF-3 and IRF-7 had a relatively modest effect on the IFN-β response in Mφ and mDC. Thus, IFN-β induction after WNV infection in myeloid cells depends on IRF-3 and IRF-7-independent transcriptional signals. This result is consistent with data showing significantly reduced but not abrogated type I IFN levels in serum of DKO mice after WNV infection. Analogously, a recent study showed residual IFN-β activity in serum after MCMV infection in independently generated DKO mice [28]. In blood, plasmacytoid DC (pDC) are believed to be primary producers of type I IFN during infection by RNA viruses [53],[54]; in pDC, IFN is induced after nucleic acid recognition by TLR7 and signaling through MyD88 to IRF-7 [14],[55]. As pDCs require IRF-7 for IFN production, yet DKO mice still produce systemic levels of IFN after WNV infection, non-pDC cell population(s) must contribute to this response. This result agrees with recent data showing no alteration of systemic type I IFN production in TLR7^−/−^ mice infected with WNV [18].

The current model for optimal IFN-β gene transcriptional depends on complete occupancy of the enhanceosome of the IFN-β gene promoter by the transcriptional factors IRF-3, IRF-7, NF-κB and ATF-2/c-Jun. Coordinate transcription factor binding enables recruitment of chromatin-remodeling proteins such as the co-activators GCN5 and CBP/p300 [23],[24]. Our data with MEF lacking IRF-3 and IRF-7 show a requirement of all individual enhanceosome constituents in efficiently regulating the IFN-β response. Consistent with this, pharmacological inhibition of the other enhanceosome constituents, NF-κB and ATF-2/c-Jun in MEF expressing IRF-3 and IRF-7 also strongly reduced the efficacy of IFN-β gene transcription. Surprisingly, this model did not apply to mDC where only functional loss of all four enhancesome components strongly diminished IFN-β gene activation. Thus, in mDC, IFN-β expression after viral infection can be induced robustly without full occupancy of the enhanceosome by the canonical transcriptional regulators. Although we do not fully understand the molecular basis for the cell-type restriction of enhanceosome occupancy and IFN-β gene induction, it is possible that in myeloid cells other transcriptional regulators can substitute for the canonical complex members to activate IFN-β gene transcription. However, other individual IRF family members (e.g., IRF-1 or IRF-8), which are known to induce the IFN-β response after viral infection or engagement of PRR in some systems [36], [56]–[58], did not have a dominant regulatory effect in the context of WNV infection. The role of IRF-2 and IRF-4 was not specifically tested as these factors have been reported as negative regulators of the type I IFN responses [59],[60]. Nonetheless, we did observe a small yet transient reduction in the IFN-β response after WNV infection in IRF-5^−/−^ mDC and had previously detected a similarly small (∼2-fold) reduction in IFN-β transcription in single-deficient IRF-7^−/−^ mDC [22]. In contrast, DKO mDC did not show altered IFN-β responses possibly because of the higher level of WNV infection in these cells.

IRF-5 was initially characterized as a transcription factor downstream of TLR-MyD88 signaling pathway that mediated induction of pro-inflammatory cytokines (e.g., IL-6 and TNF-α) excluding type I IFN [41]. More recent studies indicate that type I IFN induction in mDC after treatment with TLR4, TLR7, or TLR9 agonists is partially IRF-5-dependent [61],[62]. IRF-5 is critical for immunity against some viruses, as IRF-5^−/−^ mice have increased mortality and/or blunted systemic IFN production after infection with VSV, HSV, and NDV [38],[42]. One group recently identified a new signaling pathway involving NOD2, RIP2 and IRF-5 in modulating IFN-α/β gene induction in response to *Mycobacterium tuberculosis* [63]. Although it remains unclear precisely how IRF-5 regulates the IFN-β gene response or interacts with the enhanceosome components, its expression could account for why induction of the IFN-β response does not require complete occupancy of canonical complex constituents in myeloid cells. The generation of triple IRF-3, IRF-7 and IRF-5 knockout mice and cells may help to address this question. Alternatively, a differential level of histone acetylation of the IFN-β gene could exist in MEF and mDC. A more “relaxed” chromatin structure of the IFN-β gene in mDC might not require the coordinate recruitment of all chromatin-remodeling proteins; thus, full occupancy of the enhanceosome by transcription factors may not be a prerequisite for optimal activation of IFN-β gene transcription in this cell type.

Our results also establish that IFN-β gene activation in several cell types is largely IPS-1-dependent and likely occurs downstream of RIG-I and MDA5 recognition of WNV RNA. This data is consistent with studies in MEF, peritoneal exudates cells, and mDC with NDV, VSV, EMCV, influenza and Sendai viruses [1], [16], [64]–[66]. Similarly, a prior study with two related flaviviruses, Japanese encephalitis and Dengue viruses suggested that IFN-β gene induction in A549 lung carcinoma cells was through NF-κB and RIG-I/IRF-3-dependent pathways [67]. Our data also agrees with a previous study that observed no role for TLR3 in regulating the IFN-α/β response in mDC after WNV infection [17]. The IPS-1 dependence validates the primary role for RIG-I and/or MDA5 in sensing WNV. Although both RIG-I and MDA5 coordinately contribute to IFN and ISG induction in MEF after WNV infection [16], in mDC the RIG-I recognition pathway appears dominant ([68] and M. Suthar, S. Daffis, M. Diamond, and M. Gale, unpublished results).

**Figure 12:**
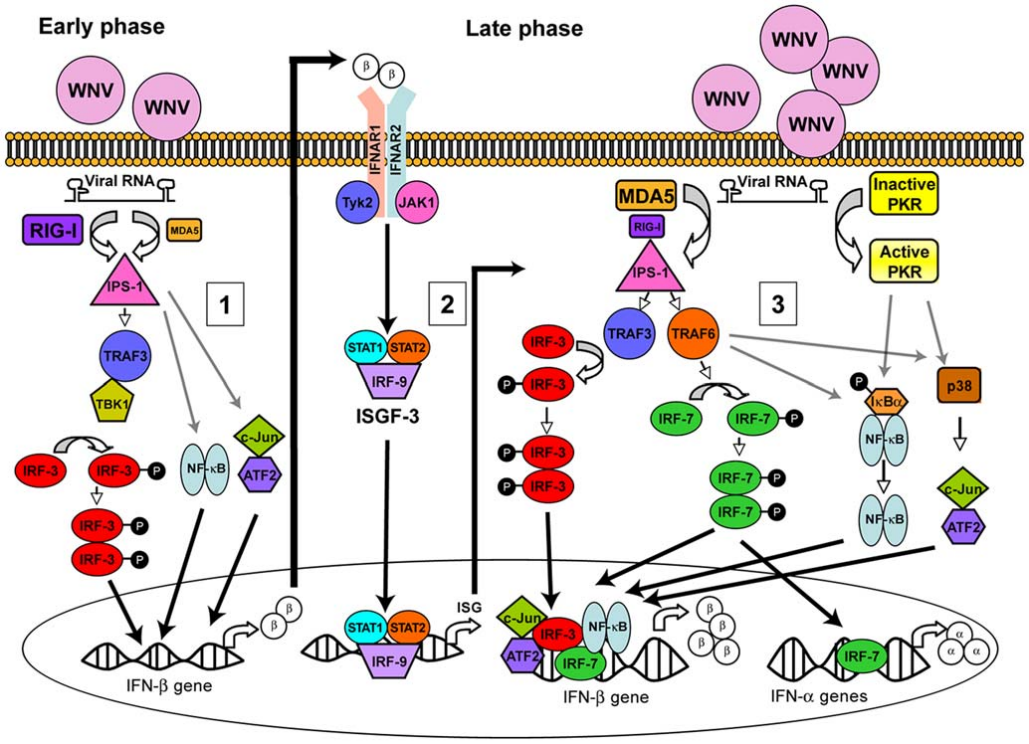
A model for detection of WNV and IFN-α/β gene activation in MEF. (1). The host through recognition of an as yet undefined viral RNA PAMP in the cytoplasm detects WNV. RIG-I acts as the primary PRR sensor for WNV during the early stages of infection. RIG-I activation promotes association with IPS-1, which leads to recruitment of TRAF3 and TBK1, and phosphorylation of IRF-3. NF-κB and ATF-2/c-Jun and the small amounts of constitutively expressed IRF-7 may also be activated via this IPS-1-dependent pathway. IRF-3, IRF-7 NF-κB, ATF-2/c-Jun translocate to the nucleus, bind the IFN-β gene promoter and promote transcription. Secretion of IFN-β by infected cells during this early phase results in autocrine and paracrine type I IFN signaling through binding of the IFN-αβR. (2). Activation of IFN-αβR results in phosphorylation of JAK1 and Tyk2, which activate STAT1 and STAT2 leading to formation of the heterotrimer ISGF3 (STAT1, STAT2 and IRF-9). Nuclear translocation and promoter binding of ISGF3 upregulates hundreds of different ISG, including IRF-7. (3). During a later phase of infection, detection of WNV in MEF also relies on MDA5 and PKR. Recruitment of TRAF3 and TRAF6 activates IRF-3 and IRF-7. NF-κB and ATF-2/c-Jun are also activated via an as yet undefined mechanism. Subsequently, IRF-3, IRF-7, NF-κB, and ATF-2/c-Jun translocate to the nucleus, bind the IFN-β gene promoter and induce optimal transcription. Induction of IFN-α genes occurs through TRAF6 and the transcriptional activation of IRF-7.

Further dissection of the IPS-1-dependent signaling pathway in MEF showed a differential role of TRAF3 and TRAF6 in regulating IFN-β responses. Based on studies with deficient MEF, TRAF3 contributes dominantly to the early phase of IFN-β production, likely by recruiting TBK1. Consistent with this, others have shown that TBK1^−/−^ MEF infected with Sendai virus have a reduced IFN-β response [69]. These results also agree with our unpublished data in IRF-3^−/−^ MEF; a deficiency of IRF-3, which is activated primarily by TRAF3 [49], results in a blunted IFN-α/β response. In contrast to TRAF3, TRAF6 had a more dominant function in sustaining the type I IFN positive feedback. Thus, after WNV infection, the type I IFN amplification loop appears mediated by signals downstream of the IFN-αβR receptor, which may include induction and/or activation of IRF-7 and TRAF6. Since TRAF3 and TRAF6 activate IRF-3, NF-κB, and p38 in MEF [49],[52], induction of the late phase of type I IFN may require these signaling adaptor molecules to activate the four components (IRF-3, IRF-7, NF-κB and ATF-2/c-Jun) of the enhanceosome. Based on the data presented here and elsewhere [16], we propose a model for host detection of WNV, signaling through IPS-1 and key adaptor molecules, and transcriptional activation of the IFN-α and β genes at early and late times after infection of MEF (**Fig 12**).

In summary, our studies demonstrate that IRF-3 and IRF-7 coordinately play essential but differential roles in vivo in protecting against WNV pathogenesis primarily by regulating the IPS-1-dependent type I IFN responses through a cell type-dependent mechanism. These studies illuminate the cell-specific complexity of IFN induction and enhance our understanding of how an effective innate response becomes activated soon after viral infection. Greater insight into the molecular mechanisms of the earliest protective antiviral immune response against WNV may provide novel strategies for therapeutic intervention against many related and unrelated viral pathogens.

## Materials and Methods

### Viruses

The WNV strain (3000.0259) was isolated in New York in 2000 [70] and passaged once in C6/36 *Aedes albopictus* cells to generate a stock (5×10^7^ PFU/ml) that was used in all experiments. Chikungunya virus (strain 142, gift of S. Higgs, UTMB) and EMCV (strain K) were propagated in C6/36 and L929, respectively.

### Mouse experiments and quantitation of viral burden

C57BL/6 wild-type mice were commercially obtained (Jackson Laboratories, Bar Harbor, ME). IFN-αβ receptor (IFN-αβR)^−/−^ congenic C57BL/6 mice were the kind gift of J. Sprent (La Jolla, CA). The congenic backcrossed IRF-3^−/−^ [37], IRF-5^−/−^ [41], and IRF-7^−/−^ [14] mice were the kind gift of T. Taniguchi (Tokyo, Japan) and provided generously by colleagues in the United States (I. Rifkin, Boston, MA and K. Fitzgerald, Worcester, MA). The IRF-1^−/−^ mice were obtained commercially (Jackson Laboratories). The IRF-8^−/−^ mDC were obtained from bone marrow of IRF-8^−/−^ mice [36] and were a generous gift of P. Tailor and K. Ozato (Bethesda, MD). The TRAF3^−/−^ and TRAF6^−/−^ MEF were kindly provided by G. Cheng (UCLA, Los Angeles, CA) and T. Mak (University of Toronto, Canada), respectively. The TBK1 MEF were obtained from B. TenOever (Mount Sinai Hospital, NY). IRF-3^−/−^× IRF-7^−/−^ DKO mice were generated after large-scale crossing and recombination in the F1 generation because of the 1 cM linkage of the two loci. DKO mice were genotyped and bred in the animal facilities of the Washington University School of Medicine, and experiments were performed with approval of the Washington University Animal Studies Committee. Eight to twelve week-old age-matched mice were used for all in vivo studies. 10^2^ PFU of WNV was diluted in Hanks balanced salt solution (HBSS) supplemented with 1% heat-inactivated fetal bovine serum (FBS) and inoculated by footpad injection in a volume of 50 µl.

### Tissue viral burden and viremia

To monitor viral dissemination in vivo, mice were infected with 10^2^ PFU of WNV by footpad inoculation and sacrificed at specific time points after inoculation. After extensive cardiac perfusion with PBS, organs were harvested, weighed, homogenized and virus was titrated by standard plaque assay as described [71]. Viral burden also was measured by analyzing WNV RNA levels using fluorogenic quantitative RT-PCR (qRT-PCR) as described [13].

### Measurement of IFN activity

#### (a) L929 bioassay

Levels of biologically active IFN in serum were measured using an EMCV L929 cytopathic effect bioassay as described [13]. Results were compared with a standard curve using recombinant mouse IFN-α (PBL Laboratories) and confirmed as IFN-specific using a neutralizing mAb (MAR-1) against the IFN-αβR [72].

#### (b) IFN-α and β mRNA by qRT-PCR

Total RNA was isolated from uninfected or WNV-infected cells at specific time points using the RNeasy kit (Qiagen). IFN-α and -β mRNA levels were measured by qRT-PCR using previously established primer sets [21]. To analyze the relative fold induction of IFN-α and β mRNA, 18S rRNA expression levels were determined in parallel for normalization using the Ct method [73].

#### (c) IFN-α and β ELISA

A commercial capture ELISA kit (PBL Laboratories) was used to measure levels of secreted IFN-α and β protein in supernatants or uninfected or WNV-infected cells.

### Primary cell culture and viral infection

#### (a) Macrophages and dendritic cells

Bone marrow derived Mφ and conventional mDC were generated as described previously [22]. Briefly, bone marrow cells were isolated from mice and cultured for seven days in the presence of macrophage colony-stimulating factor (M-CSF) (PeproTech, Inc.) to generate Mφ, or interleukin-4 (IL-4) and granulocyte-macrophage colony-stimulating factor (GM-CSF) (PeproTech, Inc.) to generate mDC. Multi-step viral growth curves were performed after infection at a multiplicity of infection (MOI) of 0.01 for Mφ or 0.001 for mDC. Supernatants were titrated by plaque assay on BHK21-15 cells. Quantitation of IFN-α and -β mRNA levels in Mφ and mDC was assessed after infection at a MOI of 0.1.

#### (b) Fibroblasts

MEF were generated from wild type and deficient 14-day-old embryos and maintained in DMEM supplemented with 10% FBS. Cells were used between passages 2 and 4 for all experiments. Multi-step virus growth curves and IFN-α and β ELISA were performed after infection at an MOI of 0.001 and 0.1, respectively.

#### (c) Cortical neurons

Primary cortical neurons were prepared from wild type and deficient 15-day-old mouse embryos as described [13]. Cortical neurons were then cultured for four days with Neurobasal medium containing B27 and L-Glutamine (Invitrogen). Multi-step virus growth curves and IFN-α and β protein quantitation were performed after infection at an MOI of 0.001 and 0.1, respectively.

### Toll-like receptor stimulation assays

mDC, generated as described above, were stimulated with TLR3 ligand (50 µg/ml poly(I∶C)) or TLR4 ligand (2 µg/ml LPS) for 24 hours. Levels of IFN-α mRNA and IFN-β protein were measured by qRT-PCR as described above.

### Western blots

Primary cells (10^6^) were lysed in RIPA buffer (10 mM Tris, 150 mM NaCl, 0.02% sodium azide, 1% sodium deoxycholate, 1% Triton X-100, 0.1% SDS, pH 7.4), with protease inhibitors (Sigma) and 1 mM okadaic acid (Sigma). Samples (30 µg) were resolved on 10% SDS-polyacrylamide gels. Following transfer, membranes were blocked with 5% non-fat dried milk overnight at 4°C. Membranes were probed with the following panel of monoclonal or polyclonal antibodies anti-WNV (Centers for Disease Control), anti-tubulin, anti-STAT1, anti-PKR, (Santa Cruz Biotechnology), and anti-mouse ISG54 (gift from G. Sen, Cleveland, Ohio). Antibodies specific to RIG-I, MDA5, IRF-3 and IRF-7 have been described [65]. Blots were incubated with peroxidase-conjugated secondary antibodies (Jackson Immunoresearch) and visualized using ECL-Plus Immunoblotting reagents (Amersham Biosciences).

### Pharmacological inhibition of NF-κB and p38/ATF-2

To evaluate the role of NF-κB and/or p38/ATF-2 in regulating the IFN-β gene expression in mDC, 2×10^5^ cells were infected with WNV and treated with 1% DMSO (diluent control), 5 µM or 10 µM of BAY 11-7082 (Calbiochem) a specific inhibitor of NF-κB and/or 5 µM or 15 µM of SB 202190 (Calbiochem), a specific inhibitor of p38/ATF-2 for 24 h. Levels of IFN-β mRNA were measured by qRT-PCR as described above. As a positive control, 2×10^5^ mDC were treated with 2 µg/ml LPS (List Biological Laboratories) for 24 h in the absence or in the presence of BAY 11-7082 and TNF-α production was monitored by ELISA (R&D Systems). Cytotoxicity of BAY 11-7082 and SB 202190 was evaluated using the Celltiter-96® Aqueous One Solution Cell proliferation Assay according to the manufacturer's instructions (Promega).

### Statistical analysis

For in vitro experiments, an unpaired two-tailed T-test was used to determine statistically significant differences. For viral burden analysis, differences in log titers were analyzed by the Mann-Whitney test. Kaplan-Meier survival curves were analyzed by the log rank test. All data were analyzed using Prism software (GraphPad Software).
